# Comparative study of allogenic and xenogeneic mesenchymal stem cells on cisplatin-induced acute kidney injury in Sprague-Dawley rats

**DOI:** 10.1186/s13287-016-0386-0

**Published:** 2016-09-01

**Authors:** Rehab H. Ashour, Mohamed-Ahdy Saad, Mohamed-Ahmed Sobh, Fatma Al-Husseiny, Mohamed Abouelkheir, Amal Awad, Doaa Elghannam, Hassan Abdel-Ghaffar, Mohamed Sobh

**Affiliations:** 1 Clinical Pharmacology Department, Faculty of Medicine, Mansoura University, Mansoura, Egypt; 2 Medical Experimental Research Center (MERC), Faculty of Medicine, Mansoura University, Mansoura, Egypt; 3 Zoology Unit-Urology and Nephrology Center, Faculty of Medicine, Mansoura University, Mansoura, Egypt; 4 Pathology Department, Mansoura University, Mansoura, Egypt; 5 Clinical Pathology Department, Faculty of Medicine, Mansoura University, Mansoura, Egypt; 6 Urology and Nephrology Center, Faculty of Medicine, Mansoura University, Mansoura, Egypt

**Keywords:** Cisplatin nephrotoxicity, Mesenchymal stem cells, Adipose-derived, Amniotic fluid-derived

## Abstract

**Background:**

The paracrine and regenerative activities of mesenchymal stem cells (MSCs) may vary with different stem cell sources. The aim of the present study is to compare the effects of MSCs from different sources on acute kidney injury (AKI) induced by cisplatin and their influence on renal regeneration.

**Methods:**

A single intraperitoneal injection of cisplatin (5 mg/kg) was used to induce AKI in 120 Sprague-Dawley rats. Rats were treated with either rat bone marrow stem cells (rBMSCs), human adipose tissue-derived stem cells (hADSCs), or human amniotic fluid-derived stem cells (hAFSCs). 5 × 10^6^ MSCs of different sources were administered through rat tail vein in a single dose, 24 hours after cisplatin injection. Within each group, rats were sacrificed at the 4th, 7th, 11th, and 30th day after cisplatin injection. Serum creatinine, BUN, and renal tissue oxidative stress parameters were measured. Renal tissue was scored histopathologically for evidence of injury, regeneration, and chronicity. Immunohistochemistry was also done using Ki67 for renal proliferative activity evaluation.

**Results:**

MSCs of the three sources were able to ameliorate cisplatin-induced renal function deterioration and tissue damage. The rat BMSCs-treated group had the lowest serum creatinine by day 30 (0.52 ± 0.06) compared to hADSCs and hAFSCs. All MSC-treated groups had nearly equal antioxidant activity as indicated by the decreased renal tissue malondialdehyde (MDA) and increased reduced glutathione (GSH) level and superoxide dismutase (SOD) activity at different time intervals. Additionally, all MSCs improved injury and regenerative scores. Rat BMSCs had the highest count and earliest proliferative activity in the renal cortex by day 7 as identified by Ki67; while, hAFSCs seem to have the greatest improvement in the regenerative and proliferative activities with a higher count of renal cortex Ki67-positive cells at day 11 and with the least necrotic lesions.

**Conclusions:**

Rat BMSCs, hADSCs, and hAFSCs, in early single IV dose, had a renoprotective effect against cisplatin-induced AKI, and were able to reduce oxidative stress markers. Rat BMSCs had the earliest proliferative activity by day 7; however, hAFSCs seemed to have the greatest improvement in the regenerative activities. Human ADSCs were the least effective in the terms of proliferative and regenerative activities.

## Background

Mesenchymal stem cells (MSCs) are an important source of tissue regeneration as they have self-renewal and multilineage differentiation potentials [1, 2]. Although adult bone marrow (BM) and adipose tissue-derived stem cells (ADSCs) are the main sources for clinical use, their use is limited because of invasive procedures in harvesting and donor age requirements [3, 4]. Therefore, new sources of MSCs, like placenta, umbilical cord blood (UCB), and amnion, have become increasingly necessary. However, these stem cell sources still require complex processing for cell isolation [5–7].

Human amniotic fluid-derived stem cells (hAFSCs), being free from ethical considerations and easy to isolate via noninvasive methods, are a fruitful new source. Additionally, they have high differentiation abilities and possess immunosuppressive potential, allowing them to be used in allogeneic settings [8, 9]. Although, hAFSCs exhibit similar characteristics to BMSCs and ADSCs, including fibroblastoid morphology, surface proteins, and differentiation potential as defined by the proposed International Society for Cellular Therapy (ISCT) criteria [10, 11], the ISCT criteria lack information of MSCs’ potential as therapeutic cell sources. Therefore, the potential of MSCs from different sources must be evaluated to select the best one for cell-based therapy in a particular disease model. Comparative studies of the therapeutic potential of hAFSCs and other MSCs sources are lacking. As the number of clinical trials and a variety of adult cells was used in regenerative therapy, cell therapeutic potential will be instantly required.

It is mandatory to realize the possible mechanisms by which MSCs induce their therapeutic effect, as this may explain the key mechanisms of tissue repair. Paracrine actions, anti-inflammatory effect, or promoting regeneration via soluble factors have been proposed [12, 13]. Although there is evidence that MSCs from diverse tissues are different, the potential benefits and mechanisms of these variously sourced MSCs remain unexplored.

Thus we sought in this study to directly compare the therapeutic potentials of MSCs of different sources including rat bone marrow stem cells (rBMSCs), hADSCs, and hAFSCs in the setting of early and late kidney injury and the role of a probable antioxidant mechanism. This may help to identify a preferable cell source for treating toxic acute kidney injury (AKI) induced by cisplatin.

## Methods

The experimental protocol was approved by the local ethics committee, Faculty of Medicine, Mansoura University.

### Agents

Cisplatin was obtained from David Bull Laboratories (Hornsby, NSW, Australia) Dulbecco’s modified Eagle’s medium (DMEM) containing 20 % fetal bovine serum (FBS) was obtained from Invitrogen (Invitrogen, Carlsbad, CA, USA).

### Stem cell isolation

#### Human adipose-derived mesenchymal stem cells

MSCs from adipose tissue were isolated as previously described by Bunnell et al. [14] with modifications. The fat was gathered under sterile conditions from liposuction surgeries and washed at least three times with phosphate-buffered saline (PBS) containing 1 % antibiotic-antimycotic solution (Thermo Fisher Scientific, Waltham, MA, USA) until all blood vessels and connective tissues appear to have been released.

Adipose tissue samples were digested in trypsin 0.125 % at 37 °C (3 mL for each 1 gm tissue) (Sigma-Aldrich, St. Louis, MO, USA) with shaking at 100 rpm for 60 minutes. Samples were observed every 15 minutes and shaken vigorously. After digestion, the trypsin activity was neutralized by adding an equal volume of DMEM containing 10 % FBS (Thermo Fisher Scientific) to the tissue sample. Then, 100-μm filters (BD Falcon, San Jose, CA, USA) were used to obtain the cell suspension and avoid the solid aggregates. After that, the samples were centrifuged at 2000 rpm for 5 minutes at room temperature. To finish the detachment of the stromal cells from the primary adipocytes, the samples were taken out of the centrifuge and shaken vigorously followed by another centrifugation step then the supernatant was removed without disturbing the cells. The pellet was resuspended in 1 mL of lysis buffer (Promega, Mannheim, Germany) to lyse red blood cells (RBCs), incubated for 10 minutes and washed with 10 mL of PBS containing 1 % antibiotic-antimycotic and was centrifuged at 2000 rpm for 5 minutes. The supernatant was aspirated and the cell pellet was resuspended in DMEM with 20 % FBS and 1 % antibiotic-antimycotic solution in a 25-cm^2^ culture flask and maintained in an incubator supplied with 5 % CO_2_ humidified atmosphere at 37 °C.

#### Human amniotic fluid-derived stem cells (hAFSCs)

Mesenchymal stem cells (MSCs) were isolated from the human amniotic fluid (hAF) of 15 women who experienced a cesarean section for breech presentation. They had given their informed consent according to the instructions of the ethics committee of the Faculty of Medicine, Mansoura University. The mean ± SD pregnancy duration (fetal age + 2 weeks) was 38 ± 1 weeks and the mean volume of the hAF samples was 10.6 ± 4.5 mL.

After the uterine muscle was opened for the cesarean section, hAF samples were collected by puncturing the membranes. MSCs were isolated from the hAF samples within 4 hours before use.

#### Bone marrow stem cells

MSCs were prepared from the bone marrow of 8-week-old male Sprague-Dawley (SD) rats according to the method used by Phinney et al. [15].

### Stem cell culture

#### Human adipose-derived mesenchymal stem cells

Non-adherent cells were removed after 1 day by repeated washes with PBS and adherent cells were further cultured in complete medium. The medium was renewed every 3 days until the monolayer of adherent cells reach 70–80 % confluence. Then, cell separation was made by trypsin-EDTA solution (0.25 %, Sigma-Aldrich) for passage 1. The number of cells was evaluated by hemocytometer and cellular viability was tested by the Trypan Blue exclusion test. Each 250–300 × 10^3^ cells were inoculated in a 75-cm^2^ culture flask and incubated at 37 °C and 5 % CO_2_. Cell cultivation was maintained up to the third passage.

#### Human amniotic fluid-derived stem cells

Samples were centrifuged at 1100 rpm for 5 minutes and all the isolated cells were cultured in six 35-mm Petri dishes containing low-glucose DMEM (Invitrogen) supplemented with 100 U/mL of penicillin, 0.1 mg/mL of streptomycin, 10 ng/mL of basic fibroblast growth factor, 10 ng/mL of epidermal growth factor (Peprotech, Rocky Hill, NJ, USA), and 20 % of FBS (Invitrogen). The medium was renewed after incubation of the cells at 37 °C with 5 % humidified CO_2_ for 7 days and the non-adhering cells were discarded.

The medium was replaced two times per week until the cells reached 70 % confluence, and then the cells were treated with 0.25 % trypsin and 1 mM EDTA (Invitrogen) for 3 minutes to release MSCs that were collected and replated in a split ratio of 1:3 under the same culture conditions.

#### Bone marrow stem cells

Bone marrow stem cells were plated in T-75 flasks in complete DMEM supplemented with 10 % FBS (Lonza, Verviers, Belgium) at 37 °C in a 5 % CO_2_ humidified atmosphere. Cells were used for experiments after the third passage.

### Flow cytometry analysis

#### Human adipose-derived mesenchymal stem cells

Mesenchymal stem cells were characterized by fluorescence-activated cell sorting (FACS) analyses using cell surface markers. The cells were stained with different fluorescently labeled monoclonal antibodies (mAb) including CD29, CD44, and CD90 (eBioscience, San Diego, CA, USA). CD34 and CD45 mAbs were used as hematopoietic lineages markers. In brief, 5 × 10^5^ cells (in 100 μL PBS/0.5 % bovine serum albumin (BSA)/2 mmol/L EDTA) were mixed with 10 μL of the fluorescently labeled mAb and incubated in the dark at 2–8 °C for 30 minutes. Washing with PBS containing 2 % BSA was done twice and the pellet was resuspended in PBS and analyzed immediately by flow cytometry. The fluorescence intensity of the cells was evaluated by EPICS-XL flow cytometry (Coulter, Miami, FL, USA).

#### Human amniotic fluid-derived stem cells

The hAF-derived stem cells were released by trypsinization at passage 3 and analyzed by flow cytometry. The cells were centrifuged (1200 rpm/5 minutes) and then set in PBS at the concentration of 1 × 10^6^/mL. The fluorescently labeled directed antibodies to CD34, CD14, CD29, CD90, CD13, CD105, and Oct4 (10 μL for each sample) were added and left for 30 minutes at room temperature. Labeled cells were thoroughly washed with PBS (2 vol) and fixed in flow buffer (1 % formaldehyde in PBS). The labeled cells were analyzed on a FACS Caliber (Becton Dickinson, Franklin Lakes, NJ, USA) by collecting 10,000 events with the Cell Quest software program (Becton Dickinson).

#### Bone marrow stem cells

MSCs were characterized using FACS analysis. Pellets of 10^5^ to 0.5 × 10^6^ cells were incubated for 30 minutes at 4 °C with fluorescently labeled antibodies particular for MSCs CD90, CD29, and CD45 (eBioscience). Cells were then washed twice, fixed in flow buffer and the fluorescence intensity was evaluated by EPICS-XL flow cytometry (Coulter).

### Colony-forming units-fibroblast

Fibroblast colony growth was examined on primary cells grown in six-well tissue culture dishes [15]. For colony-forming unit-fibroblast (CFU-F) assays, about 100 cells were plated in a 100-mm tissue culture dish (BD Falcon) in complete culture medium. Cells were incubated for 10–14 days at 37 °C in 5 % humidified CO_2_, and washed with PBS and fixed in 95 % ethanol for 5 minutes. Then, the cells were incubated for 20–30 minutes at room temperature in 0.5 % crystal violet (Sigma-Aldrich) and 95 % ethanol. After that, the plate was washed twice with distilled H_2_O. The plates were dried and the CFU-F units were counted.

### Differentiation capability of stem cells

#### Osteogenic differentiation

The harvested cells by trypsin digestion (as described above) were counted and cultured at a density of 5 × 10^4^ per well in a six-well plate; then, at 80 % confluent osteogenesis differentiation media were added to four wells: DMEM supplemented with 10 % FBS, 0.1 μM dexamethasone, 50 μM ascorbic acid, and 10 mM β-glycerol phosphate (Sigma-Aldrich). As a negative control, complete culture media was added to the other two wells [16]. The medium was renewed twice per week for 2–3 weeks. The osteogenesis differentiation potential was assessed by 40 mM Alizarin Red (pH 4.1) after fixation in 10 % neutral-buffered formalin [17].

##### Detection by Image J analysis

Twenty different digital images were analyzed using Image J 1.42 software on an appropriate threshold. These images corresponded to four different preparations of osteogenic differentiated cells and undifferentiated controls. Image J can calculate area and pixel value statistics of user-defined selections and intensity threshold objects. It can measure distances and angles. It can create density histograms and line profile plots [18].

#### Adipogenic differentiation

After the third passage, MSCs were harvested by trypsin digestion as described above; the cells were counted and cultured at a density of 10 × 10^4^ per well in a six-well plate. Then, at 100 % confluent adipogenesis differentiation media were added to four wells: DMEM supplemented with 10 % FBS, 1 μM dexamethasone, 500 μM isobutylmethylxanthine, 5 μg/mL insulin, and 200 μM indomethacin (Sigma-Aldrich). As a negative control, complete culture media was added to the other two wells, the medium was renewed twice per week for 2 weeks [16]. The differentiation potential for adipogenesis and formation of intracellular lipid droplets were assessed by Oil-red-O stain after fixation in 10 % neutral-buffered formalin [17]. Image J analysis was done as described above.

#### Chondrogenic differentiation

Harvested MSCs (6 × 10^5^ cells) were centrifuged to form a pellet on the bottom of a 15-mL polypropylene tube (BD Falcon). The micro mass was seeded in a 500-μL chondrogenic medium that consisted of 50 μg/mL ascorbic acid 2-phosphate and 1 ng/mL transforming growth factor-β_1_ (TGF-β_1_, Sigma-Aldrich) [16]. Three weeks later, cultured cell clumps were harvested, embedded in paraffin, cut into 3-μm sections, and stained for glycosaminoglycans using 0.1 % safranin O (Sigma-Aldrich). Image J analysis was done as described above.

##### Real-time quantitative reverse transcriptase-polymerase chain reaction (RT–PCR)

This aimed to test the differentiation of MSCs to adipocytes and osteocytes by expression of specific markers. Total RNA was isolated from MSCs and RT-PCR was performed as described previously [19]. Lipoprotein lipase and peroxisome proliferator activated receptor-γ (PPAR-γ) were used for adipocyte differentiation and osteocalcin gene was used for osteogenic differentiation; glyceraldehyde-3-phosphate dehydrogenase (GAPDH) was used as an internal control. The primer sequences were as follow: for lipoprotein lipase, the forward primer was 5′-GTACAGTCTTGGAGCCCATGC-3′ and the reverse primer was 5′-GCCAGTAATTCTATTGACCTTCTTGTT-3′; for PPAR-γ, the forward primer was 5′-CATACATAAAGTCCTTCCCGCTG-3′ and the reverse primer was 5′-TTGTCTGTTGTCTTTCCTGTCAAGA-3′; for osteocalcin, the forward primer was 5′-ACACTCCTCGCCCTATTG-3′ and the reverse primer was 5′-GATGTGGTCAGCCAACTC-3′; for GAPDH, the forward primer was 5′- ACAAGATGGTGAAGGTCGGTG-3′ and the reverse primer was 5′- AGAAGGCAGCCCTGGTAACC-3′.

### Animal grouping and experimental model

Rats were kept on a regular 12-hour dark/light cycle with free access to standard rat chow and tap water ad libitum. The study design and protocol was revised and approved by Mansoura local ethics committee. A total of 120 inbred female Sprague-Dawley rats (8 weeks old, weighing 200–250 g), from the Medical Experimental Research Center (MERC) at Mansoura Faculty of Medicine, were used and divided into the following groups:

Rats were randomly assigned to six equal groups (20 rats each) sacrificed at different time intervals: (I) negative control group: rats were injected with 1 mL normal saline alone; (II) positive control cisplatin-injected group: rats were injected with a single dose of cisplatin 5 mg/kg in 1 mL saline intraperintoneally (I.P.); (III) cisplatin-injected and rBMSCs-treated group: rats were injected with 0.5 mL of culture media containing 5 × 10^6^ rat BMSCs into the tail vein 1 day after cisplatin injection; (IV) cisplatin-injected and hADSCs-treated group: rats were injected with 0.5 mL of culture media containing 5 × 10^6^ human ADSCs into the tail vein 1 day after cisplatin injection; (V) cisplatin-injected and hAFSCs-treated group: rats were injected with 0.5 mL of culture media containing 5 × 10^6^ hAFSCs into the tail vein 1 day after cisplatin injection; (VI) cisplatin-injected and fresh culture media-treated group: rats were injected with 0.5 mL of culture media into the tail vein. Each main group was further subgrouped according to the time of sacrifice into four equal divisions (*n* = 5): subgroup A: rats were sacrificed at day 4 post-cisplatin injection; subgroup B: rats were sacrificed at day 7 post-cisplatin injection; subgroup C: rats were sacrificed at day 11 post-cisplatin injection; subgroup D: rats were sacrificed at day 30 post-cisplatin injection. Twenty-four-hour urine samples were collected for each rat using Nalgene metabolic cages (Nalge Nunc International Corp., Rochester, NY, USA) before sacrifice. Rats were sacrificed using an overdose of thiopental. Blood samples were then collected by syringe from the heart and immediately centrifuged at approximately 3000 g for 5 minutes, and then taken for biochemical measurements. The kidneys were removed for oxidative stress parameters and histopathological examination.

### Biochemical analysis

Kidney function tests (serum creatinine, blood urea nitrogen, creatinine clearance) were assessed; in addition, oxidative stress parameters were tested in renal tissue homogenate where malondialdehyde (MDA), superoxide dismutase (SOD), and reduced glutathione (GSH) were determined spectrophotometrically in all groups according to the methods of Ohkawa et al. [20] and Ellman [21], respectively. The following parameters were determined with the use of commercially available kits: serum and urinary creatinine (Jaffe reaction, colorimetric – kinetic; Diamond Diagnostics, Hanover, Germany), and blood urea nitrogen (BUN; Berthelot enzymatic colorimetric method, Diamond Diagnostics).

### Renal histopathology

The left kidney was prepared for histopathological examination by being perfused through the abdominal aorta in a retrograde fashion using 0.9 % saline, then 10 % neutral-buffered formalin for in situ fixation. Renal samples were coded and processed for light microscopic examination. Histopathological changes were analyzed in the different kidney regions (cortex, outer stripe of outer medulla (OSOM), inner stripe of outer medulla (ISOM), and inner medulla) using hematoxylin and eosin (H&E) stain according to a new scoring system:

***Active injury changes:*** these include necrotic tubules and interstitial infiltration by inflammatory cells. Necrotic tubules were scored according to the number of necrotic tubules counted/high power field (HPF) and scored to 1, 2, 3, and 4 according to 1–3, 4–5, 6–10, and > 10 necrotic tubules/HPF. The inflammatory cells were scored as 1, 2, and 3 corresponding to mild, moderate and severe, respectively. The maximum active injury score was 7.

***Regenerative changes:*** these include the presence of mitosis, solid cellular sheets between the tubules, intraluminal cellular proliferation forming solid tubules, tubules lined with large vesicular nuclei, and tubules lined by cells having hyperchromatic-prominent nuclei and little cytoplasm giving the luminal border a festooned appearance. Each of solid cellular sheets and solid tubules counted as 1–2, 3–5, and > 5/HPF are scored as 1, 2, and 3, respectively. Mitosis was scored as 1, 2, and 3 corresponding to 1–2, 3–5, and > 5–10/HPF, respectively. While tubules with large vesicular nuclei and tubules with basophilic-prominent nuclei get a score of 1 when present and get a score of zero if absent. The maximum regeneration score was 9.

***Chronic changes:*** these include atrophic tubules with flat lining, casts, and thick basement membrane and interstitial fibrosis; where the number of atrophic tubules/HPF of 1–5, 6–10 and > 10 are scored 1, 2, and 3, respectively. The percentages of interstitial fibrosis/HPF of 25, 25–50, 50–75, and > 75 % get scores of 1, 2, 3, and 4, respectively. The maximum chronicity score was 7.

***Renal tissue proliferative activity:*** immunohistochemistry of Ki67 monoclonal antibodies was done in cisplatin-treated rats and MSCs-treated groups. The number of Ki67-positive cells were counted per HPF and represented in each group.

### Statistical analysis

The data were analyzed using SPSS (version 16.0, SPSS Inc., Chicago, IL, USA). The data were tested for normality distribution by Kolmogorov-Smirnov test. Descriptive statistics were reported as mean ± standard deviation (SD) for normally distributed variables. One-way analysis of variance (ANOVA) followed by post hoc multiple comparisons (Bonferroni test) was used to test for significant differences between groups. Median (min-max) was used for describing nonparametric variables that were analyzed by Kruskal-Wallis (K-W) test followed by Mann-Whitney’s tests for two-group comparison. A *P* value < 0.05 was considered statistically significant.

## Results

### Stem cell culture

**Fig. 1:**
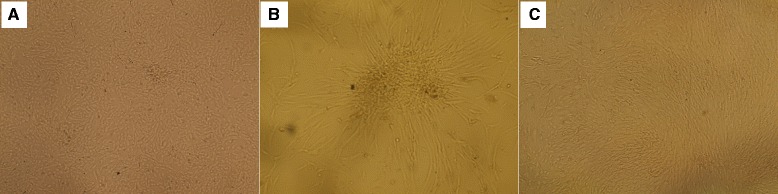
Appearance of different stem cells under inverse microscope. Under inverse microscopy, cultured rat bone marrow mesenchymal stem cells (rBMSCs, (**a**) human adipose tissue-derived mesenchymal stem cells (hADSCs, (**b**) and human amniotic fluid-derived mesenchymal stem cells (hAFSCs, (**c**) at passage 3 were morphologically defined by the fibroblast-like appearance (original magnification × 200)

One day after stem cell culture, spindle-shaped cells adherent to the tissue culture plastic flask were observed. After 5 days, spindle-shaped cells reached 80 % confluency. Morphology of the cells changed gradually with passage number as they became flatter-shaped with increasing passage number (Fig. 1a, b, c).

### Immunophenotypic FACS analysis

**Fig. 2:**
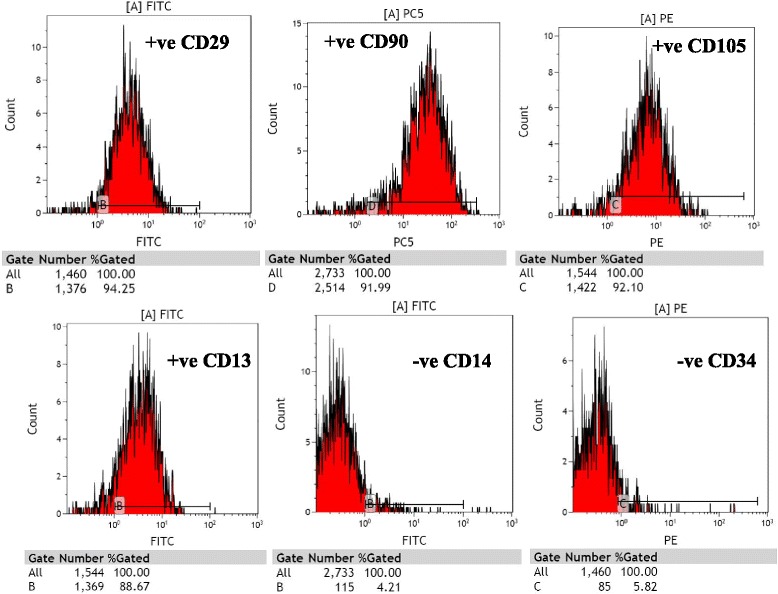
Analysis of hADSCs with flow cytometry. Flow cytometry analysis of hADSCs revealed that their expression of surface antigens such as CD29, CD90, CD105, and CD13 was positive; but, CD14 and CD34 was negative (passage 3)

**Fig. 3:**
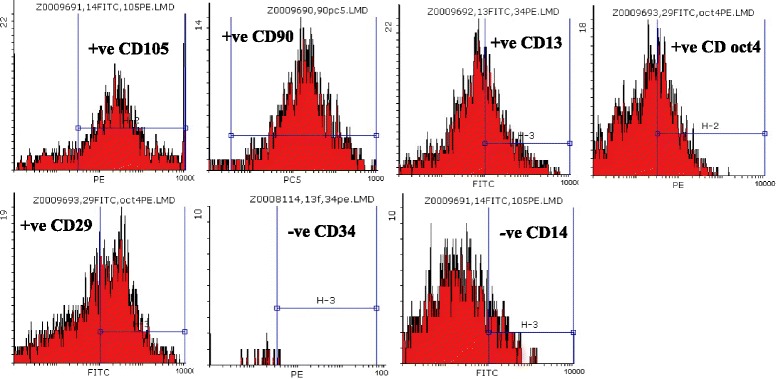
Analysis of hAFSCs with flow cytometry. Flow cytometry analysis of hAFSCs revealed that their expression of surface antigens such as CD105, CD90, CD13, Oct4, and CD29 was positive; but CD34 and CD14 was negative (passage 3)

**Fig. 4:**
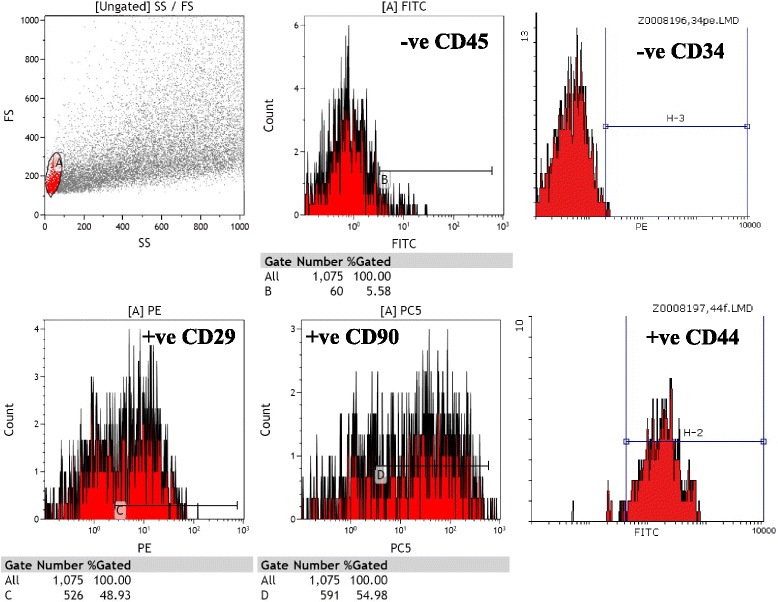
Analysis of rBMSCs with flow cytometry. Flow cytometry analysis of rat bone marrow mesenchymal stem cells revealed that their expression of surface antigens such as CD29, CD90, and CD44 was positive; but CD45 and CD34 was negative (passage 3)

Cultures of SCs were analyzed for expression of cell-surface markers. hADSCs revealed their expression of surface antigens CD29 (94 %), CD90 (92 %), CD105 (92 %), and CD13 (89 %) were strongly positive; but, CD14 (4 %) and CD34 (6 %) were negative (passage 3, Fig. 2). hAFSCs were positive for CD105 (79.5 %), CD90 (79.1 %), CD29 (50 %), CD13 (36.5 %), and Oct4 (31.4 %); but, negative for CD14 (11.3 %) and CD34 (15 %) (passage 3, Fig. 3). Moreover, rat BMSCs were negative for the hematopoietic lineage marker (CD34 and CD45) and positive for CD44 (93.7 %), CD29 (48.9 %), and CD90 (54.9 %) (passage 3, Fig. 4).

### CFU-F

**Fig. 5:**
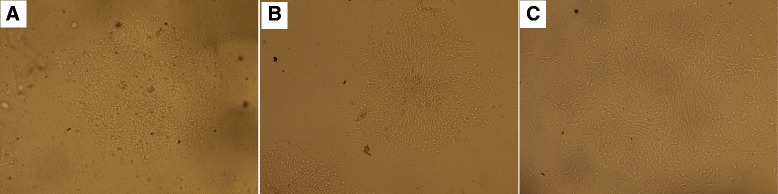
Photomicrographs of different stem cells’ colonies after 1 week of culture. Photomicrographs of rat bone marrow mesenchymal stem cells (rBMSCs, (**a**) human adipose tissue-derived mesenchymal stem cells (hADSCs, (**b**) and human amniotic fluid-derived mesenchymal stem cells (hAFSCs, (**c**) after 1 week of culture grew in colonies that contained heterogeneous small spindle-shaped fibroblastoid cells and more rounded cells (original magnification × 100)

CFU-F assay is a suitable tool for evaluating the proliferation and clonogenic capacity of the cells expanded in culture (third passage). The colony number of 100 SCs per 100-mm tissue culture dish was around 38 ± 1 (Fig. 5a, b, c).

### Stem cell differentiation capability

#### Osteogenic differentiation

**Fig. 6:**
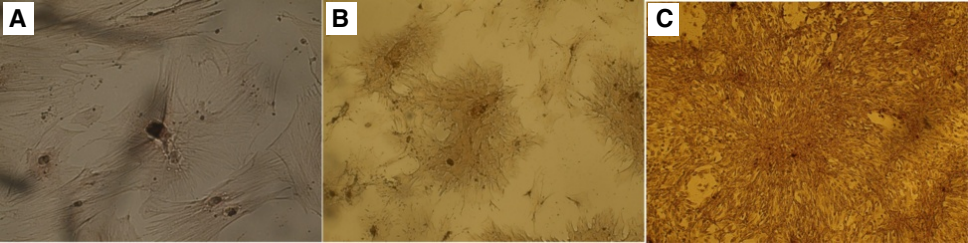
Photomicrographs showing osteogenic differentiation potential of different stem cells. Photomicrographs of rat bone marrow mesenchymal stem cells (rBMSCs, (**a**) human adipose tissue-derived mesenchymal stem cells (hADSCs, (**b**) and human amniotic fluid-derived mesenchymal stem cells (hAFSCs, (**c**) showing differentiation potential toward osteoblasts as indicated by the formation of calcium-rich hydroxyapatite detected with Alizarin Red and appearing as irregular red-orange (original magnification × 200)

Based on Image J analysis and spectrophotometric analysis, SCs tended to differentiate into osteocytes. Image J analysis showed a 3.4 ± 0.5 fold increase of differentiated versus undifferentiated MSCs (Fig. 6a, b, c).

#### Adipogenic differentiation

**Fig. 7:**
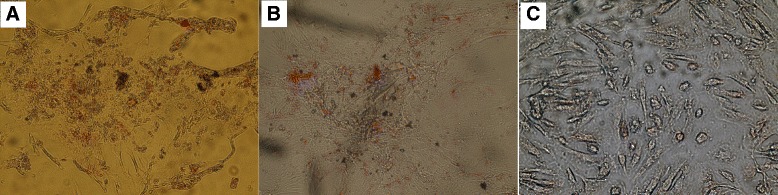
Photomicrographs showing adipogenic differentiation potential of different stem cells. Photomicrographs of rat bone marrow mesenchymal stem cells (rBMSCs, (**a**) human adipose tissue-derived mesenchymal stem cells (hADSCs, (**b**) and human amniotic fluid-derived mesenchymal stem cells (hAFSCs, (**c**) showing adipocyte differentiation potential visualized by highly refractive intracellular lipid vacuoles and droplets appear as cherry red spheres within the cells by Oil-red-O staining (original magnification × 100)

Based on Image J analysis and spectrophotometric analysis, SCs tended to differentiate into adipocytes. Image J analysis showed a 2.6 ± 0.7 fold increase of differentiated versus undifferentiated MSCs (Fig. 7a, b, c).

#### Chondrogenic differentiation

**Fig. 8:**
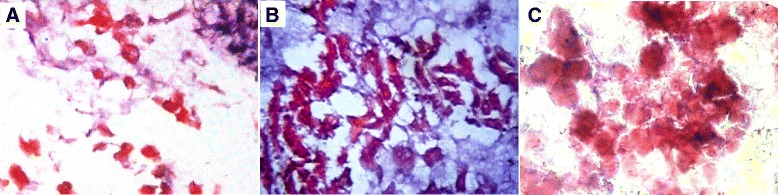
Photomicrographs showing chondrogenic differentiation potential of different stem cells. Photomicrographs of rat bone marrow mesenchymal stem cells (rBMSCs, (**a**) human adipose tissue-derived mesenchymal stem cells (hADSCs, (**b**) and human amniotic fluid-derived mesenchymal stem cells (hAFSCs, (**c**) showing chondrogenic differentiation potential visualized by formation of glycosaminoglycans with a red color (original magnification × 100)

Based on Image J analysis and spectrophotometric analysis, SCs tended to differentiate into chondrocytes. Image J analysis showed a 3.3 ± 0.6 fold increase of differentiated versus undifferentiated MSCs (Fig. 8a, b, c).

#### Stem cell differentiation by real-time quantitative RT–PCR

**Fig. 9:**
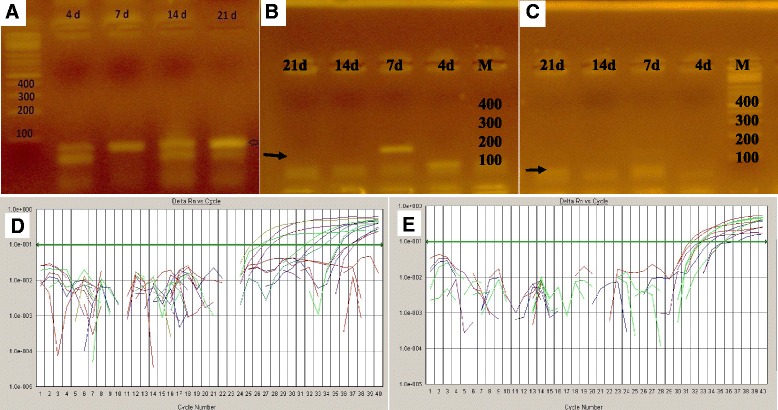
Gel electrophoresis and RT-PCR gene expression of different stem cells. Gel electrophoresis for lipoprotein lipase showed that: lane M (DNA marker) made DNA ladder and lane 4d, 7d, 14d, and 21d showed bands present at suspected size and proved the formation of lipoprotein lipase (*arrow*) of rat bone marrow mesenchymal stem cells (BMSCs) (**a**), human adipose tissue-derived mesenchymal stem cells (hADSCs) (**b**), and human amniotic fluid-derived mesenchymal stem cells (hAFSCs) (**c**). RT- PCR gene expression showed about a tenfold increase in gene expression of PPAR-γ during adipocytic differentiation (**d**) and osteocalcin during osteocytic differentiation of MSCs (**e**) as indicated by the curves above the threshold *green line*

Rat BMSCs in adipogenic culture conditions expressed lipoprotein lipase at different time intervals (after 4, 7, 14, and 21 days) as evidenced by gel electrophoresis (Fig. 9a) and PPAR-γ, a transcription factor known to be involved in the control of adipocytic differentiation, as shown by about a tenfold increase in gene expression by RT-PCR (Fig. 9d). Also, hADSCs and hAFSCs in adipogenic culture conditions expressed lipoprotein lipase at the same different time intervals (Fig. 9b, c respectively). In addition, PPAR-γ gene expression was evidenced by RT-PCR for hADSCs and hAFSCs (Fig. 9d). Rat BMSCs, hADSCs, and hAFSCs in osteogenesis culture conditions expressed osteocalcin at the same different time intervals as evidenced by about a tenfold increase in gene expression by RT-PCR (Fig. 9e).

### Effect of SCs on cisplatin-induced renal impairment: serum creatinine, BUN, and creatinine clearance

In this study, I.P. injection of 5 mg/kg cisplatin led to significant increase in serum creatinine and BUN (*P* < 0.001), which were high on the 4th and 7th day, declined at the 11th day and stabilized at 30th day to a value higher than the baseline. Meanwhile, intravenous (IV) injection of 5 × 10^6^ SC of different sources, 24 hours after cisplatin injection, strongly prevented renal function deterioration at different time intervals as reflected by significantly lower serum creatinine and BUN values with respect to cisplatin-treated and fresh culture media-treated rats intravenously (*P* < 0.001 on the 4th and 7th day). No significant differences exist between the SCs-treated groups, as shown in Table 1.

**Table 1:** Effect of different types of stem cells on biochemical measurements (*n* = 20 and *n* = 5 for day subgroups, mean ± SD)

	Group I	Group II	Group III	Group IV	Group V	Group VI
	negative control	cisplatin ‘CDDP’-treated	cisplatin + rBMSCs-treated	cisplatin + hADSCs-treated	cisplatin + hAFSCs-treated	cisplatin + DMEM culture media-treated
Sr. Cr. (mg/dL)						
Day 4	0.40 ± 0.12	1.92 ± 0.03^a^	0.92 ± 0.05^ab^	0.97 ± 0.02^ab^	1.02 ± 0.08^ab^	1.88 ± 0.10^acde^
Day 7	0.37 ± 0.12	1.61 ± 0.05^a^	0.79 ± 0.04^ab^	0.87 ± 0.06^ab^	0.85 ± 0.07^ab^	1.63 ± 0.03^acde^
Day 11	0.40 ± 0.11	1.00 ± 0.07^a^	0.67 ± 0.1^ab^	0.72 ± 0.05^ab^	0.70 ± 0.06^ab^	1.02 ± 0.10^acde^
Day 30	0.39 ± 0.10	0.77 ± 0.03 ^a^	0.52 ± 0.06^ab^	0.58 ± 0.06^ab^	0.58 ± 0.05^ab^	0.81 ± 0.04^acde^
BUN (mg/dL)						
Day 4	18.6 ± 1.14	85.6 ± 1.67^a^	32.0 ± 1.58^ab^	30.2 ± 0.83^ab^	34.4 ± 1.14^ab^	85.4 ± 1.14^acde^
Day 7	18.2 ± 1.48	55.2 ± 1.30^a^	27.2 ± 1.30^ab^	27.8 ± 1.30^ab^	28.0 ± 1.22^ab^	53.8 ± 0.84^acde^
Day 11	17.2 ± 1.48	32.3 ± 1.48^a^	24.2 ± 0.83^ab^	24.2 ± 1.30^ab^	24.4 ± 0.89^ab^	32.4 ± 1.51^acde^
Day 30	18.0 ± 1.58	26.4 ± 0.55^a^	22.2 ± 0.83^ab^	22.4 ± 0.89^ab^	23.2 ± 0.84^ab^	26.2 ± 0.83^acde^
Cr. Cl. (mL/min/100 gm)						
Day 4	1.62 ± 0.44	0.007 ± 0.001^a^	0.021 ± 0.005^a^	0.021 ± 0.006^a^	0.018 ± 0.006^a^	0.008 ± 0.001^a^
Day 7	1.78 ± 0.55	0.014 ± 0.001^a^	0.052 ± 0.008^a^	0.048 ± 0.001^a^	0.045 ± 0.001^a^	0.012 ± 0.002^a^
Day 11	1.86 ± 0.56	0.05 ± 0.01^a^	0.07 ± 0.01^a^	0.06 ± 0.01^a^	0.06 ± 0.007^a^	0.05 ± 0.013^a^
Day 30	1.76 ± 0.55	0.36 ± 0.17^a^	0.92 ± 0.23^a^	0.89 ± 0.15^a^	1.00 ± 0.14^ab^	0.46 ± 0.26^a^

Values are expressed as mean ± SD. A significant difference (*P* < 0.05) between different groups was done by Bonferroni post hoc for multiple comparisons

*rBMSCs* rat bone marrow stem cells, *hADSCs* human adipose tissue-derived stem cells, *hAFSCs* human amniotic fluid-derived stem cells, *DMEM* Dulbecco’s modified Eagle’s medium, *Sr. Cr*. serum creatinine, *BUN* blood urea nitrogen, *Cr. Cl*. creatinine clearance

^a^Significant difference vs. negative control group ^b^Significant difference vs. cisplatin-treated group ^c^Significant difference vs. cisplatin + rBMSCs-treated group ^d^Significant difference vs. cisplatin + hADSCs-treated group ^e^Significant difference vs. cisplatin + hAFSCs-treated group

Regarding creatinine clearance, it was markedly reduced by cisplatin injection (*P* < 0.001 on the 4th, 7th, 11th, and 30th day). Therapy with MSCs from different sources attenuated these changes with no significant differences between SCs-treated groups, as shown in Table 1.

### Effect of SCs on MDA, GSH levels, and SOD activities in renal tissue homogenate

As shown in Table 2, renal tissue homogenate MDA level was significantly increased in cisplatin-injected rats compared to the negative control group at different time intervals (*P* < 0.001). A significant decrease in tissue MDA was noted after IV injection of 5 × 10^6^ SC of different sources at days 4, 7, 11, and 30 when compared with cisplatin-treated rats and rats given fresh culture media intravenously (*P* < 0.001).

**Table 2:** Effect of different types of stem cells on renal tissue oxidative stress parameters (*n* = 20 and *n* = 5 for day subgroups, mean ± SD)

	Group I	Group II	Group III	Group IV	Group V	Group VI
	negative control	cisplatin ‘CDDP’-treated	cisplatin + rBMSCs-treated	cisplatin + hADSCs-treated	cisplatin + hAFSCs-treated	cisplatin + DMEM culture media-treated
MDA (nmol/gm tissue)						
Day 4	14.6 ± 1.61	66.5 ± 2.83^a^	34.7 ± 4.95^ab^	37.0 ± 3.75^ab^	34.7 ± 6.72^ab^	66.6 ± 2.86^acde^
Day 7	14.9 ± 1.50	64.9 ± 3.93^a^	24.5 ± 4.98^ab^	28.0 ± 2.58^ab^	26.1 ± 2.13^ab^	66.9 ± 2.29^acde^
Day 11	15.4 ± 1.63	35.5 ± 3.33^a^	18.4 ± 0.93^b^	18.3 ± 2.20^b^	17.8 ± 1.78^b^	34.8 ± 2.84^acde^
Day 30	15.1 ± 1.62	30.1 ± 4.61^a^	16.2 ± 0.87^b^	18.7 ± 2.32^b^	17.6 ± 2.29^b^	28.1 ± 1.58^acde^
GSH (mmol/gm tissue)						
Day 4	5.40 ± 0.25	0.26 ± 0.03^a^	0.78 ± 0.04^ab^	0.81 ± 0.11^ab^	0.76 ± 0.02^ab^	0.26 ± 0.02^acde^
Day 7	5.44 ± 0.23	0.54 ± 0.03^a^	2.65 ± 0.15^ab^	2.72 ± 0.17^ab^	2.64 ± 0.36^ab^	0.52 ± 0.03^acde^
Day 11	5.55 ± 0.17	1.49 ± 0.33^a^	3.36 ± 0.14^ab^	3.68 ± 0.13^ab^	3.55 ± 0.15^ab^	1.32 ± 0.30^acde^
Day 30	5.32 ± 0.31	1.92 ± 0.03^a^	5.05 ± 0.12^b^	4.29 ± 0.18^abc^	3.98 ± 0.43^abc^	1.91 ± 0.05^acde^
SOD (U/g of tissue)						
Day 4	20.36 ± 1.70	2.98 ± 0.19^a^	7.64 ± 0.37^ab^	6.87 ± 0.39^ab^	6.93 ± 0.42^ab^	2.94 ± 0.23^acde^
Day 7	19.92 ± 1.39	6.24 ± 0.42^a^	10.85 ± 0.38^ab^	10.31 ± 0.36^ab^	10.69 ± 0.33^ab^	6.22 ± 0.45^acde^
Day 11	19.88 ± 1.40	9.98 ± 0.32^a^	15.46 ± 0.32^ab^	15.80 ± 0.25^ab^	15.73 ± 0.29^ab^	10.0 ± 0.25^acde^
Day 30	20.22 ± 1.70	15.3 ± 0.36^a^	18.26 ± 0.17^ab^	18.62 ± 0.38^ab^	18.74 ± 0.15^b^	15.6 ± 0.49^acde^

Values are expressed as mean ± SD. A significant difference (*P* < 0.05) between different groups was done by Bonferroni post hoc for multiple comparisons

*rBMSCs* rat bone marrow stem cells, *hADSCs* human adipose tissue-derived stem cells, *hAFSCs* human amniotic fluid-derived stem cells, *DMEM* Dulbecco’s modified Eagle’s medium, *MDA* malondialdehyde, *GSH* reduced glutathione, *SOD* superoxide dismutase

^a^Significant difference vs. negative control group ^b^Significant difference vs. cisplatin-treated group ^c^Significant difference vs. cisplatin + rBMSCs-treated group ^d^Significant difference vs. cisplatin + hADSCs-treated group ^e^Significant difference vs. cisplatin + hAFSCs-treated group

On the other hand, renal tissue homogenate GSH level and SOD activity were significantly decreased in cisplatin-injected rats compared to the negative control group at different time intervals (*P* < 0.001). In SCs-treated groups, the mean values of renal tissue homogenate GSH level and SOD activity were significantly increased at different time intervals compared to the cisplatin-treated rats and rats given fresh culture media intravenously (*P* < 0.001), as shown in Table 2.

### Effect of SCs on cisplatin-induced renal histopathological changes and score

The various histopathological changes and related scores (Table 3) observed in kidney tissue at different time intervals are summarized below:

**Table 3:** Active injury score, regeneration score, and chronicity score in OSOM of different experimental groups (*n* = 20 and *n* = 5 for days subgroups, mean ± SD)

	Group I	Group II	Group III	Group IV	Group V	Group VI
	negative control	cisplatin ‘CDDP’-treated	cisplatin + rBMSCs-treated	cisplatin + hADSCs-treated (*n* = 20)	cisplatin + hAFSCs-treated	cisplatin + DMEM culture media-treated
Active injury						
Day 4	0.0 (0.0–0.0)	7.0 (7.0–7.0)^a^	3.0 (2.0–3.0)^ab^	3.0 (2.0–3.0)^ab^	3.0 (2.0–3.0)^ab^	6.0 (6.0–7.0)^abcde^
Day 7	0.0 (0.0–0.0)	6.0 (6.0–6.0)^a^	3.0 (3.0–3.0)^ab^	3.0 (2.0–3.0)^ab^	3.0 (2.0–3.0)^ab^	6.0 (6.0–6.0)^abcde^
Day 11	0.0 (0.0–0.0)	4.0 (4.0–4.0)^a^	1.0 (1.0–1.0)^ab^	1.0 (1.0–1.0)^ab^	1.0 (1.0–1.0)^ab^	6.0 (5.0–6.0)^abcde^
Day 30	0.0 (0.0–0.0)	4.0 (4.0–4.0)^a^	1.0 (0.0–1.0)^b^	1.0 (1.0–1.0)^b^	1.0 (1.0–1.0)^ab^	3.0 (3.0–3.0)^abcde^
Regeneration						
Day 4	0.0 (0.0–0.0)	0.0 (0.0–0.0)	4.0 (4.0–5.0)^ab^	4.0 (4.0–5.0)^ab^	4.0 (3.0–5.0)^ab^	0.0 (0.0–0.0)^cde^
Day 7	0.0 (0.0–0.0)	0.0 (0.0–0.0)	6.0 (5.0–6.0)^ab^	6.0 (6.0–6.0)^ab^	7.0 (6.0–7.0)^abc^	0.0 (0.0–0.0)^cde^
Day 11	0.0 (0.0–0.0)	1.0 (1.0–2.0)^a^	8.0 (7.0–8.0)^ab^	8.0 (7.0–9.0)^ab^	8.0 (8.0–8.0)^ab^	1.0 (1.0–2.0)^acde^
Day 30	0.0 (0.0–0.0)	3.0 (3.0–3.0)^a^	9.0 (8.0–9.0)^ab^	8.0 (7.0–9.0)^ab^	8.0 (7.0–9.0)^ab^	3.0 (3.0–3.0)^acde^
Chronicity						
Day 4	0.0 (0.0–0.0)	3.0 (3.0–3.0)^a^	2.0 (1.0–2.0)^ab^	2.0 (1.0–2.0)^ab^	2.0 (2.0–2.0)^ab^	3.0 (3.0–3.0)^acde^
Day 7	0.0 (0.0–0.0)	4.0 (4.0–4.0)^a^	2.0 (2.0–3.0)^ab^	2.0 (2.0–2.0)^ab^	2.0 (2.0–2.0)^ab^	4.0 (4.0–5.0)^acde^
Day 11	0.0 (0.0–0.0)	5.0 (5.0–5.0)^a^	2.0 (2.0–2.0)^ab^	2.0 (2.0–2.0)^ab^	2.0 (2.0–2.0)^ab^	5.0 (5.0–5.0)^acde^
Day 30	0.0 (0.0–0.0)	3.0 (3.0–4.0)^a^	2.0 (2.0–2.0)^ab^	2.0 (2.0–2.0)^ab^	2.0 (2.0–2.0)^ab^	3.0 (3.0–3.0)^acde^

Values are expressed as mean ± SD. A significant difference (*P* < 0.05) between different groups was done by Kruskal-Wallis test

*OSOM* outer strip of outer medulla, *rBMSCs* rat bone marrow stem cells, *hADSCs* human adipose tissue-derived stem cells, *hAFSCs* human amniotic fluid-derived stem cells, *DMEM* Dulbecco’s modified Eagle’s medium

^a^Significant difference vs. negative control group ^b^Significant difference vs. cisplatin-treated group ^c^Significant difference vs. cisplatin + rBMSCs-treated group ^d^Significant difference vs. cisplatin + hADSCs-treated group ^e^Significant difference vs. cisplatin + hAFSCs-treated group

#### Day 4

**Fig. 10:**
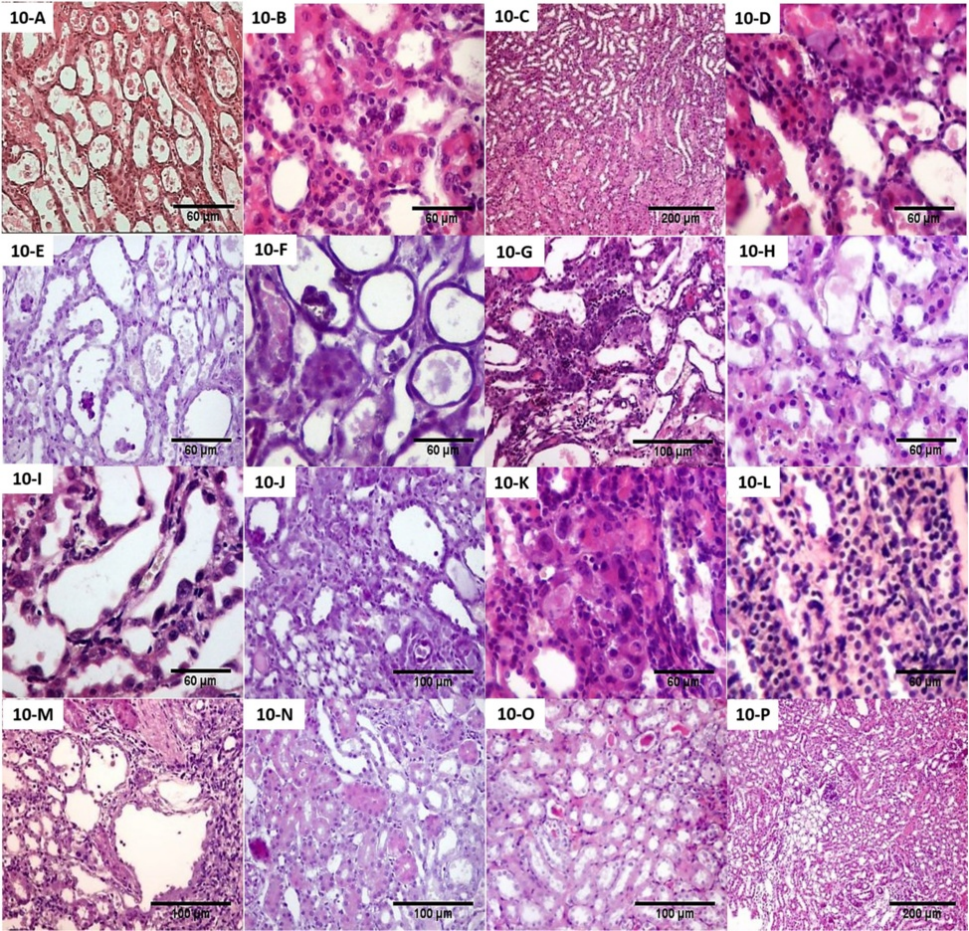
Renal pathological changes of experimental groups at different time points. Pathological changes in cisplatin-injected rats sacrificed at day 4 (**a**) showed marked degenerative changes and atrophic tubules in OSOM (H&E, ×200). Pathological changes in cisplatin-injected and rat bone marrow stem cells (rBMSCs)-treated rats (**b**), human adipose tissue-derived stem cells (hADSCs)-treated rats (**c**), and human amniotic fluid-derived stem cells (hAFSCs)-treated rats (**d**) sacrificed at day 4 showed regenerative changes in the OSOM in the form of many regenerating tubules lined by large cells with large hyperchromatic nuclei with few solid sheets (H&E, B and D × 200, C × 40). Pathological changes in cisplatin-injected rats sacrificed at day 7 (**e**) showed combined degenerative and regenerative changes (H&E, ×200). Pathological changes in cisplatin-injected and rBMSCs-treated rats (**f**), hADSCs-treated rats (**g**), and hAFSCs-treated rats (**h**) sacrificed at day 7 showed less marked degenerative changes and many regenerative changes in the OSOM in the form of many interstitial solid sheets (H&E, F and H × 200, G × 100). Pathological changes in cisplatin-injected rats sacrificed at day 11 (**i**) showed more marked degenerative changes and mild interstitial round cell infiltrate (H&E, ×200). Pathological changes in cisplatin-injected and rBMSCs-treated rats (**j**), hADSCs-treated rats (**k**), and hAFSCs-treated rats (**l**) sacrificed at day 11 showed more regenerative changes, few necrotic tubules, and interstitial round cell infiltrates (H&E, J × 100, and K and L × 200). Pathological changes in cisplatin-injected rats sacrificed at day 30 (**m**) showed mild peritubular and perivascular fibrosis (H&E, ×100). Pathological changes in cisplatin-injected and rBMSCs-treated rats (**n**), hADSCs-treated rats (**o**), and hAFSCs-treated rats (**p**) sacrificed at day 30 showed less chronic changes as regard the tubular atrophy and the renal fibrosis (H&E, N and O × 100, P × 40)

Renal sections obtained from cisplatin-injected rats (group II, Fig. 10a) and cisplatin-injected rats treated with culture media through the tail vein (group VI) showed marked degenerative changes mainly in OSOM. These changes ranged from tubular cell vacuolar degeneration up to complete tubular necrosis with apoptosis and shedding of most of the tubular epithelial cells. Mild regenerative changes were also present in OSOM in the form of some regenerating tubules lined by large cells with prominent nucleoli and occasional mitotic figures. No solid sheets were detected.

In SCs-injected groups (group III, Fig. 10b; group IV, Fig. 10c; group V, Fig. 10d), regenerative changes were markedly detected in the OSOM and ISOM in the form of many regenerating tubules lined by large cells with large hyperchromatic active nuclei with few solid sheets.

#### Day 7

Histopathological features observed in cisplatin-injected (group II, Fig. 10e) and cisplatin-injected rats treated with culture media through the tail vein (group VI) were present mainly in the OSOM in the form of combined degenerative and regenerative changes. The degenerative changes were predominant in all studied fields in this group and varied from tubular cell vacuolar degeneration up to complete tubular necrosis with pyknosis and shedding of tubular epithelial cells. The regenerative changes were detected in about 20 % of all fields examined in the group and varied from tubular cell enlargement, mitosis, and interstitial solid sheet formation.

Renal tissue of cisplatin-injected rats treated with SCs of different sources (Fig. 10f, g, h) showed less marked degenerative changes observed mainly in OSOM. The degenerative changes were the same as described previously but less than what were seen on day 4. Regenerative changes also detected in both outer and inner medulla in the form of many interstitial solid sheets and tubules lined by large cells with prominent nucleoli and with occasional mitosis.

#### Day 11

More marked degenerative changes were detected in renal sections obtained from cisplatin-injected rats (group II, Fig. 10i) and cisplatin-injected rats treated with culture media through the tail vein (group VI). In addition, there was mild interstitial round cell infiltrate. Regenerative changes were also detected and varied from tubular cell enlargement with regenerative atypia, mitosis, and interstitial solid sheet formation.

Renal tissue obtained from cisplatin-injected rats treated with SCs of different sources via the tail vein (Fig. 10j, k, l) revealed few necrotic tubules with epithelial shedding and tubular dilatation. More regenerative changes were detected in the form of solid sheets having prominent bulging nuclei, mitosis, and regenerating tubules. The interstitium was the seat of focal round cell infiltration.

#### Day 30

Renal sections obtained from cisplatin-injected rats (group II, Fig. 10m) and cisplatin-injected and treated with culture media through the tail vein (group VI) revealed occasional regenerating large tubules in the OSOM lined by large cells with prominent nucleoli and occasional mitotic figures. There is mild peritubular and perivascular fibrosis (about 5–10 % of all fields).

Cisplatin-injected rats treated with SCs of different sources via the tail vein and showed significantly less chronic changes as regard the tubular atrophy and the renal fibrosis when compared with the cisplatin-treated group (Fig. 10n, o, p).

Collectively, SCs-treated groups of different sources (groups III, IV, V) showed more regenerative changes as early as the 4th day with less tubular necrosis and atrophy than in cisplatin-injected rats (group II) and cisplatin-injected rats treated with culture media through the tail vein (group VI). In addition, the SCs maintained a renoprotective effect and ameliorated renal injury till the 30th day.

#### Renal tissue proliferative activity

**Fig. 11:**
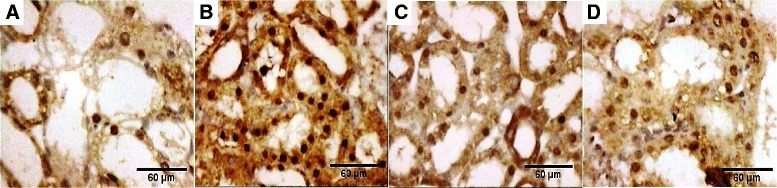
Proliferating cells/HPF identified by Ki67 immunohistochemistry in OSOM at day 11 of different experimental groups. Proliferating cells/HPF identified by Ki67 immunohistochemistry in OSOM at day 11 of cisplatin-injected rats (**a**), cisplatin-injected and rat bone marrow stem cells (rBMSCs)-treated rats (**b**), cisplatin-injected and human adipose-derived tissue stem (hADSCs)-treated rats (**c**), and cisplatin-injected and human amniotic fluid-derived stem cells (hAFSCs)-treated rats (**d**) as positive regenerating tubules and some scattered cells representing interstitial epithelial solid sheets (magnification × 200)

The immunohistochemistry of Ki67 was used for evaluation of the renal tissue proliferative activity. The results capitulate in Table 4 and Fig. 11a, b, c, d. The rat BMSCs-treated group (group III) had the earliest and the highest proliferative potential, especially in the renal cortex. The renal sites of the evolution of Ki67-positive cells throughout the experimental groups were only the mid and deep cortex (day 4 and 7), then started to appear in the OSOM at day 11 in the form of positive regenerating tubules and some scattered cells as interstitial epithelial solid sheets. However, ISOM and inner medulla were negative. At day 30, the mid and deep cortex showed Ki67-positive cells in the proximal tubules; while, OSOM, ISOM, and inner medulla were negative.

**Table 4:** Number of proliferating cells/HPF identified by Ki67 in the renal cortex and OSOM of different experimental groups

	Group I	Group II	Group III	Group IV	Group V	Group VI
	negative control	cisplatin ‘CDDP’-treated	cisplatin + rBMSCs-treated	cisplatin + hADSCs-treated (*n* = 20)	cisplatin + hAFSCs-treated	cisplatin + DMEM culture media-treated
Renal cortex						
Day 4	6.0 ± 1.58	8.6 ± 1.14	20.8 ± 2.77^ab^	18.8 ± 1.30^ab^	18.8 ± 3.34^ab^	8.4 ± 1.14^cde^
Day 7	9.0 ± 1.58	13.8 ± 2.38	26.4 ± 3.05^ab^	17.8 ± 1.92^ac^	16.4 ± 5.59^ab^	12.2 ± 1.92^c^
Day 11	7.4 ± 1.34	16.4 ± 3.05	18.0 ± 3.16^a^	33.8 ± 5.54^abc^	42.4 ± 6.69^abc^	25.0 ± 4.12^ae^
Day 30	7.4 ± 1.14	36.0 ± 2.91^a^	29.6 ± 2.88^ab^	25.8 ± 2.95^ab^	26.2 ± 3.03^ab^	34.4 ± 3.05^ade^
OSOM						
Day 4	6.0 ± 1.58	6.8 ± 1.92	22.2 ± 2.28^ab^	21.6 ± 2.40^ab^	23.4 ± 3.64^ab^	14.8 ± 3.96^abcde^
Day 7	8.0 ± 1.58	12.4 ± 2.07	29.6 ± 1.67^ab^	30.0 ± 3.16^ab^	23.4 ± 7.30	21.8 ± 2.86^abc^
Day 11	7.0 ± 1.58	13.8 ± 2.86	29.6 ± 5.55^ab^	26.8 ± 3.27^ab^	26.2 ± 2.38^ab^	23.6 ± 3.84^ab^
Day 30	6.8 ± 0.83	37.4 ± 5.27^a^	30.6 ± 7.92^a^	31.6 ± 2.70^a^	25.0 ± 4.12^a^	37.6 ± 3.64^ae^

Values are expressed as mean ± SD. A significant difference (*P* < 0.05) between different groups was done by Bonferroni post hoc for multiple comparisons

*HPF* high power field, *OSOM* outer strip of outer medulla, *rBMSCs* rat bone marrow stem cells, *hADSCs* human adipose tissue-derived stem cells, *hAFSCs* human amniotic fluid-derived stem cells, *DMEM* Dulbecco’s modified Eagle’s medium

^a^Significant difference vs. negative control group ^b^Significant difference vs. cisplatin-treated group ^c^Significant difference vs. cisplatin + rBMSCs-treated group ^d^Significant difference vs. cisplatin + hADSCs-treated group ^e^Significant difference vs. cisplatin + hAFSCs-treated group

## Discussion

With the evolution of the stem cell era, BMSCs, hADSCs, and hAFSCs have been available sources of cell-based therapy. Different research groups had identified and compared cellular morphology, surface markers, and differentiation abilities of stem cells from several sources [11, 22]. In this study, we directly compared the therapeutic potentials of MSCs of different sources including rBMSCs, hADSCs, and hAFSCs in the setting of cisplatin-induced early and late kidney injury. Prior to that, we reported a full characterization of different MSCs used. FACS analysis showed all MSCs were positive for expression of a cluster of differentiation (CD) of a mesenchymal nature (CD29, CD44, and CD90) and negative for the hematopoietic lineage markers (CD34 and CD45). Moreover, the differentiation abilities of MSCs of different sources were also established. Cells showed multipotent differentiation potential into the three lineages of osteogenesis, chondrogenesis, and adipogenesis that is a standard for defining MSCs. This was evaluated by using special stain for each lineage; Alizarin Red stain for osteogenesis, safranin O stain for chondrogenesis, and Oil-red-O stain for adipogenesis that stains cytoplasmic lipid droplets.

In addition, hAFSCs showed similar MSC properties with either rBMSCs or hADSCs in their morphology and surface markers. In the third passage, hAFSCs exhibited a spindle-shaped morphology analogous to that of rBMSCs and hADSCs. Human AFSCs were comparable to rBMSCs and hADSCs in their mesenchymal differentiating potentials. Human ADSCs and AFSCs showed MSC features as defined by the ISCT minimum criteria: a spindle shape, multilineage differentiation, and surface marker expression. Kern et al. [22] showed that ADSCs were preferable as they hold the highest frequency of MSCs; however, hAFSCs seemed to be more expandable. Such studies aimed for more detailed description of stem cell biology of various sources.

The direction to use hAFSCs in cell-based therapy is its better senescence patterns which vary between MSC sources [23]. Zaim et al. [24] observed that senescence was age-dependent. In addition, it was demonstrated that neonatal MSCs showed no sign of cellular senescence over long-term culture [25]. The biological properties of neonatal MSCs were determined to be different from adult MSCs. One study showed a significant decrease in stem cell characteristics with advancing donor age in humans MSCs [26]. In addition, hAFSCs showed great expansion potential and senescence retardation than ADSCs and BMSCs in spite of their longer culture time [27]. Further studies of MSC senescence are required to address these issues.

A major concern that remains to be uncovered is determining which cell source is most appropriate and effective in a specific disease model. Here, we directly compared the therapeutic potentials of rBMSCs, hADSCs, and hAFSCs in the context of cisplatin-induced early and late kidney injury and the role of a probable antioxidant mechanism. MSCs of the three sources were able to ameliorate cisplatin-induced renal function deterioration and tissue damage. The rat BMSCs-treated group had the lowest serum creatinine by day 30 compared to hADSCs and hAFSCs. Moreover, all MSCs-treated groups had nearly equal antioxidant activities at different time intervals. To quantitatively test the paracrine properties of various MSCs, oxidative stress markers were assessed in renal tissue homogenate of different groups. Increased MDA free radical and decreased GSH level and SOD activity occurred with cisplatin toxicity. On the other hand, the level of these markers was significantly changed in all MSC treatment groups.

It is necessary to recognize the mechanism of MSC-induced tissue recovery since this may explain the several aspects of tissue repair. Earlier studies showed that MSCs had the capacity to home to the site of injury and differentiate into specific cell lineages through a mechanical effect [28]. In our data, MSCs from the three different sources injected intravenously had a specific mechanism underlying their therapeutic benefit and this could be related to the paracrine role of secreted growth factors and cytokines [29] that modify the cellular microenvironment, decreasing oxidative stress, limiting damage, and enhancing tissue repair. The early administration of a single dose of stem cells permits the speculation of an antioxidant paracrine-protective mechanism for stem cells of the three sources against the acute nephrotoxicity of cisplatin.

Assessment of renal tissue proliferative activity by the evolution of Ki67-positive cells means that the migrating stem cells (whether extrarenal or intrarenal from the niche of the inner medulla) aggregated but remained quiescent in the earlier periods (day 4) till they reached the interstitium as aggregates of peritubular solid sheets in the later period (day 11) where they began to colonize the necrotic tubules and started to divide actively and regenerate the damaged tissues. After complete regeneration (day 30), all compartments of the kidney returned quiescent Ki67-negative except the cortex. We speculated that the Ki67-positive cells were migrating extrarenal stem cells by their time-course appearance in kidney tissue. Inconsistent with our results, one detailed study demonstrated a time-linked evidence of incorporation of extrarenal BMSCs during regeneration in a renal ischemia rat model [30]. Contrarily to this speculation, exogenous stem cells may modulate the renal microenvironment through paracrine action that enhances or mobilizes interstitial renal residents [31].

To the best of our knowledge, this is the first study to directly compare the therapeutic potentials of MSCs of different sources including rBMSCs, hADSCs, and hAFSCs in the setting of early and late kidney injury and the role of a probable antioxidant mechanism. The limitation of the present study is that the rat BMSCs used were not completely pure as noticed from the results of the FACS (CD29, 48.9 % and CD90, 54.9 %). These cells did not exactly meet one of the minimal criteria of MSCs. Still, other criteria such as plastic adherence and differentiation are fulfilled [10].

## Conclusions

In summary, we suggest that various MSCs tested have comparable therapeutic potential against cisplatin-induced acute kidney injury. All types of stem cells led to significant improvement in all tested biochemical and histopathological parameters. The beneficial effects appeared as early as the 4th-day subgroups evaluations. rBMSCs seem to have significantly greater improvement in the renal function tests. It has the highest antioxidant activity. However, hAFSCs seem to have the greatest improvement in the regenerative and proliferative activities with a higher count of Ki67-positive cells on day 11 with the least necrotic lesions (Table 4, Fig. 11d). We believe that primitive hAFSCs have biological advantages in comparison to other adult sources, making these cells a useful tool for clinical applications of cell therapy.

## Abbreviations

AKI: acute kidney injury
BSA: bovine serum albumin
BUN: blood urea nitrogen
CD: cluster of differentiation
CFU-F: colony-forming unit-fibroblast
DMEM: Dulbecco’s modified Eagle’s medium
FACS: fluorescence-activated cell sorting
FBS: fetal bovine serum
GAPDH: glyceraldehyde-3-phosphate dehydrogenase
GSH: reduced glutathione
hADSCs: human adipose tissue-derived stem cells
hAFSCs: human amniotic fluid-derived stem cells
HPF: high power field
ISCT: International Society For Cellular Therapy
ISOM: inner stripe of outer medulla
mAb: monoclonal antibodies
MDA: malondialdehyde
MSCs: mesenchymal stem cells
OSOM: outer stripe of outer medulla
PBS: phosphate-buffered saline
PPAR-γ: peroxisome proliferator-activated receptor-γ
rBMSCs: rat bone marrow stem cells
RT-PCR: real-time quantitative reverse transcriptase-polymerase chain reaction
SD: Sprague-Dawley
SOD: superoxide dismutase
TGF-β1: transforming growth factor- β1
UCB: umbilical cord blood

## References

[1] Jiang Y, Jahagirdar BN, Reinhardt RL, Schwartz RE, Keene CD, Ortiz-Gonzalez XR, Reyes M, Lenvik T, Lund T, Blackstad M (2002). Pluripotency of mesenchymal stem cells derived from adult marrow. Nature.

[2] Caplan AI (2007). Adult mesenchymal stem cells for tissue engineering versus regenerative medicine. J Cell Physiol.

[3] Mosna F, Sensebe L, Krampera M (2010). Human bone marrow and adipose tissue mesenchymal stem cells: a user’s guide. Stem Cells Dev.

[4] Alves H, van Ginkel J, Groen N, Hulsman M, Mentink A, Reinders M, van Blitterswijk C, de Boer J (2012). A mesenchymal stromal cell gene signature for donor age. PLoS One.

[5] Karamzadeh R, Eslaminejad MB, Aflatoonian R (2012). Isolation, characterization and comparative differentiation of human dental pulp stem cells derived from permanent teeth by using two different methods. J Vis Exp.

[6] Lu LL, Liu YJ, Yang SG, Zhao QJ, Wang X, Gong W, Han ZB, Xu ZS, Lu YX, Liu D (2006). Isolation and characterization of human umbilical cord mesenchymal stem cells with hematopoiesis-supportive function and other potentials. Haematologica.

[7] Han K, Lee JE, Kwon SJ, Park SY, Shim SH, Kim H, Moon JH, Suh CS, Lim HJ (2008). Human amnion-derived mesenchymal stem cells are a potential source for uterine stem cell therapy. Cell Prolif.

[8] Oh W, Kim DS, Yang YS, Lee JK (2008). Immunological properties of umbilical cord blood-derived mesenchymal stromal cells. Cell Immunol.

[9] Reinisch A, Bartmann C, Rohde E, Schallmoser K, Bjelic-Radisic V, Lanzer G, Linkesch W, Strunk D (2007). Humanized system to propagate cord blood-derived multipotent mesenchymal stromal cells for clinical application. Regen Med.

[10] Dominici M, Le Blanc K, Mueller I, Slaper-Cortenbach I, Marini F, Krause D, Deans R, Keating A, Prockop D, Horwitz E (2006). Minimal criteria for defining multipotent mesenchymal stromal cells. The International Society for Cellular Therapy position statement. Cytotherapy.

[11] Bosch J, Houben AP, Radke TF, Stapelkamp D, Bunemann E, Balan P, Buchheiser A, Liedtke S, Kogler G (2012). Distinct differentiation potential of “MSC” derived from cord blood and umbilical cord: are cord-derived cells true mesenchymal stromal cells?. Stem Cells Dev.

[12] Caplan AI, Dennis JE (2006). Mesenchymal stem cells as trophic mediators. J Cell Biochem.

[13] Singer NG, Caplan AI (2011). Mesenchymal stem cells: mechanisms of inflammation. Annu Rev Pathol.

[14] Bunnell BA, Flaat M, Gagliardi C, Patel B, Ripoll C (2008). Adipose-derived stem cells: isolation, expansion, and differentiation. Methods.

[15] Phinney DG, Kopen G, Isaacson RL, Prockop DJ (1999). Plastic adherent stromal cells from the bone marrow of commonly used strains of inbred mice: variations in yield, growth, and differentiation. J Cell Biochem.

[16] Rombouts WJ, Ploemacher RE (2003). Primary murine MSC show highly efficient homing to the bone marrow but lose homing ability following culture. Leukemia.

[17] Peister A, Mellad JA, Larson BL, Hall BM, Gibson LF, Prockop DJ (2004). Adult stem cells from bone marrow (MSCs) isolated from different strains of inbred mice vary in surface epitopes, rates of proliferation, and differentiation potential. Blood.

[18] Collins TJ (2007). Image J, for microscopy. Biotechniques.

[19] Van Roeyen CR, Ostendorf T, Denecke B, Bokemeyer D, Behrmann I, Strutz F, Lichenstein HS, LaRochelle WJ, Pena CE, Chaudhuri A, Floege J (2006). Biological responses to PDGF-BB versus PDGF-DD in human mesangial cells. Kidney Int.

[20] Ohkawa H, Ohishi N, Yagi K (1979). Assay of lipid peroxides in animal tissues by thiobarbituric acid reaction. Anal Biochem.

[21] Ellman GL (1959). Tissue sulphydryl groups. Arch Biochem Biophys.

[22] Kern S, Eichler H, Stoeve J, Kluter H, Bieback K (2006). Comparative analysis of mesenchymal stem cells from bone marrow, umbilical cord blood, or adipose tissue. Stem Cells.

[23] Cheng H, Qiu L, Ma J, Zhang H, Cheng M, Li W, Zhao X, Liu K (2011). Replicative senescence of human bone marrow and umbilical cord derived mesenchymal stem cells and their differentiation to adipocytes and osteoblasts. Mol Biol Rep.

[24] Zaim M, Karaman S, Cetin G, Isik S (2012). Donor age and long-term culture affect differentiation and proliferation of human bone marrow mesenchymal stem cells. Ann Hematol.

[25] Hass R, Kasper C, Bohm S, Jacobs R (2011). Different populations and sources of human mesenchymal stem cells (MSC): a comparison of adult and neonatal tissue-derived MSC. Cell Commun Signal.

[26] Kretlow JD, Jin YQ, Liu W, Zhang WJ, Hong TH, Zhou G, Baggett LS, Mikos AG, Cao Y (2008). Donor age and cell passage affects differentiation potential of murine bone marrow-derived stem cells. BMC Cell Biol.

[27] Jin HJ, Bae YK, Kim M, Kwon S-J, Jeon HB, Choi SJ, Kim SW, Yang YS, Oh W, Chang JW (2013). Comparative analysis of human mesenchymal stem cells from bone marrow, adipose tissue, and umbilical cord blood as sources of cell therapy. Int J Mol Sci.

[28] Charbord P (2010). Bone marrow mesenchymal stem cells: historical overview and concepts. Hum Gene Ther.

[29] Doorn J, Moll G, Le Blanc K, van Blitterswijk C, de Boer J (2012). Therapeutic applications of mesenchymal stromal cells: paracrine effects and potential improvements. Tissue Eng Part B Rev.

[30] Vansthertem D, Caron N, Declèves AE, Cludts S, Gossiaux A, Nonclercq D, Toubeau G (2008). Label-retaining cells and tubular regeneration in postischemic kidney. NDT.

[31] Yeagy BA, Cherqui S (2011). Kidney repair and stem cells: a complex and controversial process. Pediatr Nephrol.

